# TOUCH^®^ Prosthesis for Thumb Carpometacarpal Joint Osteoarthritis: A Prospective Case Series

**DOI:** 10.3390/jcm10184090

**Published:** 2021-09-10

**Authors:** Stefan M. Froschauer, Matthias Holzbauer, Julian A. Mihalic, Oskar Kwasny

**Affiliations:** 1 Department for Trauma Surgery and Sport Traumatology, Med Campus III, Kepler University Hospital Linz, Krankenhausstrasse 3, 4020 Linz, Austria; julian.mihalic@kepleruniklinikum.at (J.A.M.); Oskar.kwasny@kepleruniklinikum.at (O.K.); 2 Faculty of Medicine, Johannes Kepler University Linz, Altenbergerstraße 69, 4020 Linz, Austria; 3 Microsurgical Training and Research Center (MAZ), Kepler University Hospital GmbH, Krankenhausstrasse 3, 4020 Linz, Austria

**Keywords:** arthroplasty, dual mobility prosthesis, thumb carpometacarpal joint, total joint replacement, TOUCH prosthesis

## Abstract

The dual mobility concept currently represents the newest generation of thumb carpometacarpal prostheses. The aim of this study was to evaluate the short-term outcomes of TOUCH^®^ prosthesis. From September 2019 to July 2020, 40 prosthesis were implanted in 37 patients suffering from symptomatic stage III osteoarthritis. All included patients with a median age of 57.7 (IQR: 13.6) finished the systematic follow-up regimen (4, 8, 16 weeks, 6, and 12 months postoperatively). All parameters significantly improved (*p* < 0.0001) compared to the preoperative status. At 1 year follow-up, median DASH Scores decreased from 54 (IQR 22) to 12 (IQR 28) and pain levels improved from 8 (IQR 2) to 1 (IQR 2). Moreover, key-pinch strength increased from 3.8 (2.0) to 5.8 (2.5), while palmar abduction, radial abduction, and opposition also significantly improved. 35/37 patients were satisfied with the functional outcomes. We observed 10 complications, of which 6 were tendon-related issues, and 2 were due to an inappropriate choice of neck size. We could detect one dislocation but no evidence of cup loosening, tilting or subsidence in any patient. Despite the occurrence of some complications, we recommend implantation of this prosthesis type due to favorable clinical and radiological performance.

## 1. Introduction

**Figure 1:**
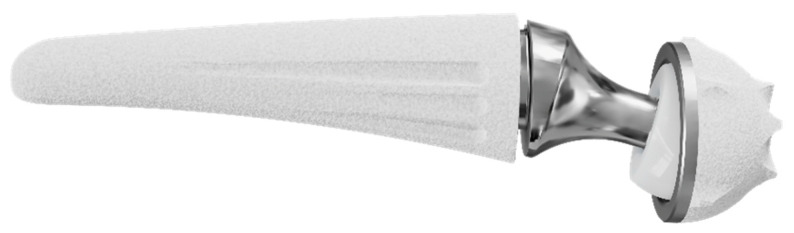
TOUCH^®^ dual mobility thumb CMC prosthesis (KeriMedical, Les Acacias, Switzerland).

The thumb carpometacarpal (CMC) joint is the second most common location in the hand affected by primary osteoarthritis (OA) [1]. If conservative treatment for this degenerative condition is exhausted, a wide range of surgical options are available. Although traditional surgical methods, i.e., trepeziectomy (with or without ligament reconstruction) or arthrodesis, are still broadly used, the most recent literature on the thumb CMC joint focuses on the performance of total joint arthroplasty (TJA) [2,3]. Various implant types pursue the identical objective, namely to achieve satisfactory pain relief, improve key-pinch strength, maintain motion, prevent thumb shortening and simultaneously limiting postoperative recovery time [3]. However, a widespread use of TJA is currently hampered by a persistent association with its initial difficulties, i.e., high complication rates caused by aseptic loosening, subsidence, subluxation and dislocation [4]. To overcome these initial difficulties of first-generation cemented prostheses [5], cementless prostheses with various cup and stem designs as well as with both metal-metal and metal-polyethylene bearings have been developed [2]. The most recent innovation in thumb CMC TJA is the integration of dual mobility. This concept was initially developed by Gilles Bousquet in 1976 for total hip arthroplasty: The association of two articulating surfaces in the prosthesis cup intends to reduce the dislocation rate and increases the angular displacement to decrease the mechanic loads on the fixation points [6]. TOUCH^®^ prosthesis (KeriMedical, Les Acacias, Switzerland) adopts this properties for the thumb CMC joint (see Figure 1).

The present study aims to assess clinical and radiographical outcomes of TOUCH^®^ prosthesis 1 year postoperatively in a prospective case series, while DASH Scores were defined as the primary outcome parameter.

## 2. Materials and Methods

Between September 2019 and July 2020, patients suffering from symptomatic thumb CMC OA with radiographic Eaton–Littler stage III [7] and failed non-surgical treatment were included in this prospective case series. The lead author (FSM) enrolled patients which were eligible to participate in the present study. After written informed consent was obtained by all participants, patients underwent implantation of the TOUCH^®^ prosthesis by a single Level IV surgeon [8]. All patients agreed to clinical follow-up after 4, 8, and 16 weeks as well as both clinical and radiographical follow-up appointments 6 months, 12 months, 2 years, 4 years and 10 years postoperatively. This study represents a prospective case series with per-protocol analysis reporting outcomes 1 year after surgery; hence, a complete follow-up protocol was a prerequisite for inclusion in the current evaluation. DASH scores were defined as primary outcome variable.

Patients suffering from bilateral symptomatic thumb CMC OA were eligible to be included. Any history of trauma (e.g., Bennett’s fracture-dislocation or Rolando fracture), concomitant OA of the wrist, especially the scapho-trapezio-trapezoid joint, any previous thumb CMC surgery, or diagnosed rheumatoid arthritis were defined as exclusion criteria.

This trial was approved by the Institutional Review Board of Ethical Commission of Johannes Kepler University Linz (Approval Number: 1094/2018) and adheres to the WMA Declaration of Helsinki.

### 2.1. Surgical Technique

All procedures were performed by a single Level IV experienced hand surgeon [8] assisted by a resident under regional brachial plexus and using a tourniquet.

The TOUCH^®^ prosthesis was introduced by Bruno Lussiez and Pascal Ledoux in 2015 [9,10]. The prosthesis consists of an anatomical stem made of titanium alloy, available in five different sizes, in which a stainless-steel neck (available in 6, 8 and 10 mm height) with a 4 mm head either in a straight or 15° offset version is inserted. The dual mobility mechanism is warranted by a polyethylene inset, which is pre-assembled on the head. The inner surface with a 4 mm diameter articulates with the prosthesis’s head the outer surface of 7 mm diameter by articulating with the stainless-steel cup implanted in the trapezium. Thus, this prosthesis consists of two concentric articulations: a smaller one between the stainless-steel head and a polyethylene inset as well as a larger one between the polyethylene inset and the trapezial cup. This spherical cup with five anti-rotation fins and spiked crown is available in 9 and 10 mm diameter. Osteointegration of both the metacarpal stem and the trapezial cup is promoted by a press-fit mechanism, and a double-layer porous titanium and hydroxyapatite coating.

**Figure 2:**
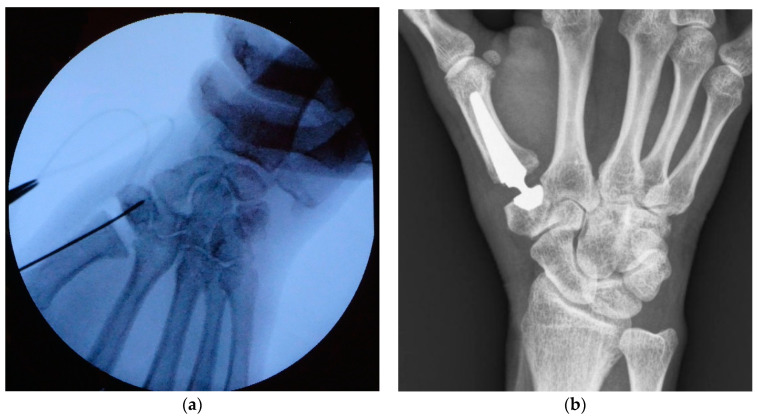
Intraoperative fluoroscopy showing Kirschner wire positioning before reaming (**a**); X-ray of TOUCH^®^ prosthesis taken at 1 year follow-up (**b**).

For prosthesis implantation, a technique described by Regnard [11] was applied in our institution. Particular care was taken while positioning the Kirschner in the center of the trapezium wire under fluoroscopy control (in at least two plains) before reaming was started (see Figure 2a). Postoperative care included 3 weeks of immobilization in a splint, followed by 6 weeks of hand therapy.

### 2.2. Clinical and Radiographical Evaluation

Upon inclusion in the study, date of birth, sex, side of the operation, and the dominant hand were recorded. Patients completed the Disabilities of the Arm, Shoulder and Hand (DASH) questionnaire [12]. Pain was assessed while asking the patients to specify their average pain levels in everyday life caused by the affected thumb CMC joint on a visual analogue scale (VAS) ranging from 0 (no pain) to 10 (worst imaginable pain). Moreover, preoperative data acquisition included key-pinch strength measurement of the affected as well as contralateral thumb using a dynamometer, and range of motion (ROM) assessment: radial and palmar abduction via radius-metacarpal angle [13] as well as distance (in cm) between the thumb tip and the fifth metacarpophalangeal joint while the patients opposed their thumbs maximally.

After 4, 8, and 16 weeks, patients were examined on how symptoms had changed as compared with the preoperative condition, using a Likert scale with five items as follows: 1 equaled “much better”, 2 represented “better”, 3 meant an “unaltered status”, whereas 4 was “worse”, and 5 was “much worse”.

After 6 and 12 months, patients were called in for a follow-up examination including assessment of DASH score, VAS, key-pinch strength, and ROM. Radiographs were taken in two planes (anteroposterior and lateral) at each visit (see Figure 2b). They were screened for dislocation, implant loosening (radiolucent areas around the prosthesis components), cup tilting, subsidence, and adjacent joint OA. At our final follow-up after 12 months, subjective satisfaction was evaluated by asking if the patients would undergo prosthesis implantation again. Moreover, every occurring complication, patient’s subjective complaints, reoperation, or revision surgery at any time after prosthesis implantation was noted.

### 2.3. Statistical Methods

Descriptive statistics are presented as median (interquartile range (IQR)) due to the non-normal distribution of our data. The side difference (Δ) in key-pinch strength was calculated via subtracting the value of the operated hand from the contralateral one. Bilateral cases are excluded from this assessment.

Comparative testing was undertaken using the repeated measures ANOVA for normal parameters and the Friedman test for non-normal distribution. If these tests revealed a significant result, pairwise comparisons were conducted using Dunn–Bonferroni post hoc tests and adjusted significance values are reported. Box plots with asterisks indicating the *p*-values resulting from the pairwise comparisons were generated for DASH Scores, VAS, and key-pinch strength values.

A value of *p* < 0.05 was considered significant.

## 3. Results

The inclusion criteria were met by 37 patients, of whom 3 received bilateral surgery, resulting in 40 thumbs assessed in the present study. Patient demographics are displayed in Table 1. No patient was lost to follow-up.

**Figure 3:**
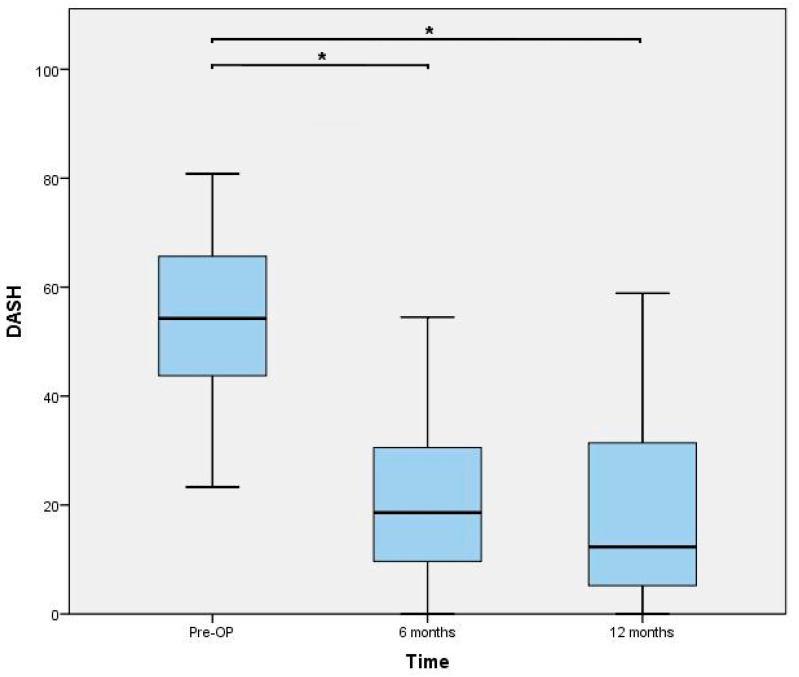
Disabilities of the Arm, Shoulder and Hand (DASH) score outcomes. * indicating a *p*-value < 0.0001.

**Figure 4:**
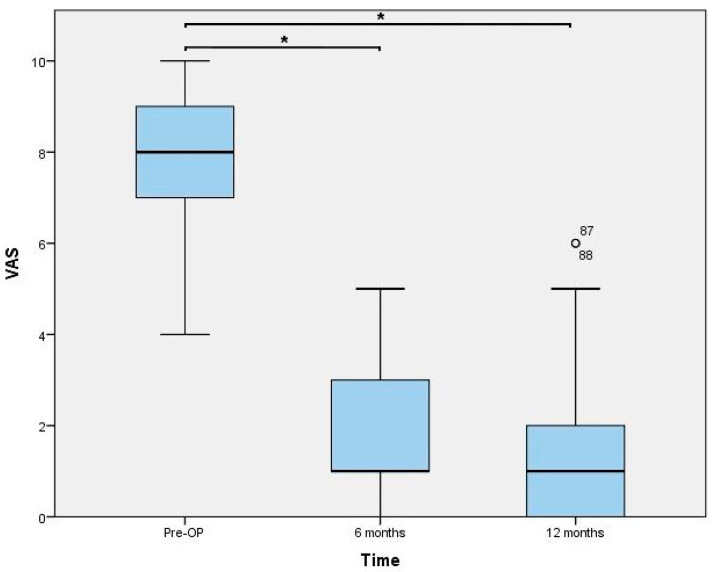
Visual analogue scale (VAS) values for pain. * indicating a *p*-value < 0.0001. Statistical outliers are presented using a small circle.

**Figure 5:**
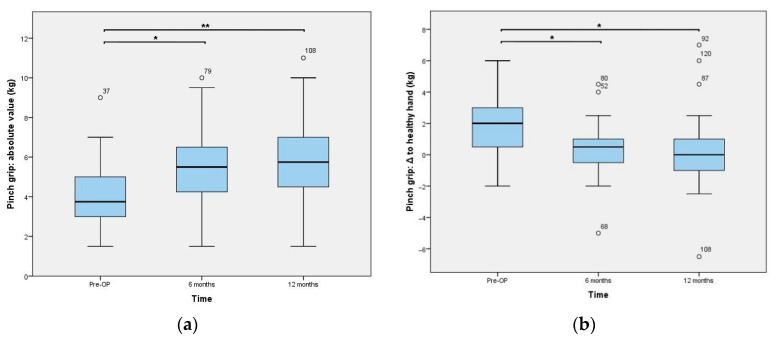
Key-pinch strength outcomes: (**a**) absolute values and (**b**) difference between the value of the operated hand and the contralateral one, which was solely calculated in unilateral cases (n = 34). * is indicating a *p*-value of 0.0001 and ** represents <0.0001. Statistical outliers are presented using a small circle.

Functional outcomes assessed 6 and 12 months postoperatively are presented in Figure 3, Figure 4 and Figure 5. The results of the ROM measurements are summarized in Table 2. The Friedman test resulted in a *p*-value < 0.0001 for all parameters. In a further step, all pairwise comparisons showed significant improvements.

The Likert scale assessing the subjective change of symptoms resulted in median values (IQR) of 2 (0), 2 (1), and 1 (1) after 4, 8, and 16 weeks. Although the Friedman test resulted in a significant result (*p* = 0.001), pairwise comparisons showed no significant result (3W vs. 6W: *p* = 0.94; 3W vs. 12W: *p* = 0.09).

At 1 year follow-up, 35 out of 37 patients (95%) were satisfied with the procedure and the functional outcomes, respectively, while 2 patients (5%) were not content with the result.

Regarding complications, there was one case of extraprosthetic dislocation. However, in total we observed 10 complications (25%), of which two de Quervain’s tendosynovitis and one irritation of the superficial branch of the radial nerve could be solved conservatively. Five complications required reoperation: one wound revision surgery due to surgical site infection, two extensor indicis transfers due to extensor pollicis longus (EPL) tendon ruptures (3 and 7 months postoperatively), one de Quervain’s release, and one trigger thumb release. Moreover, two complications required revision surgery: one patient showed dislocation of the prosthesis 7 days postoperatively, caused by a too short prosthesis neck. Another patient presented with painful limitation of ROM 5 months postoperatively. In this case, revision surgery revealed a very tightly implanted prosthesis. The underlying cause of the patients’ symptoms could subsequently be solved via exchanging the neck.

Radiographic work-up showed no evidence of cup loosening, tilting or subsidence in any patient. At final follow-up, all cups were totally integrated into bone.

## 4. Discussion

The present study represents a single-surgeon experience with the TOUCH^®^ thumb CMC prosthesis. This dual-mobility implant resulted in a significant reduction of functional impairment related to thumb CMC OA. Thus, our primary outcome parameter, i.e., the DASH score value, could be decreased by 42 points which exceeds the minimal clinically important difference of 10 points [14]. Moreover, our secondary outcome parameters corroborate this finding because pain levels decrease to a median VAS level of 1, and key-pinch strength approached to the level of the contralateral hand leading to a subjective satisfaction rate of 95%.

The primary objective of the dual mobility design is to decrease the dislocation rate [15], a feared complication in unconstrained ball and socket prostheses [16]. Mechanically, this is achieved by increasing the diameter of the articulating head [17]. Because the pre-assembly of the insert warrants a certain protection against dislocation of the 4 mm head, this principle is achieved in the articulation between outer surface of the insert (7 mm diameter) and the trapezial cup. Moreover, Lussiez et al. report that the smaller articulation contributes additional 34° of ROM to the outer articulation, leading to a total value of 117° [9,15]. Thus, not only is better patient mobility achieved, but this feature also intends to reduce (shear) strain on the cup and to prevent neck-cup contact [18,19]. Another characteristic of this prosthesis type is the metal-on-polyethylene bearing, which currently seems the best component for TJA. In previous reports, metal-on-metal prosthesis, e.g., the Elektra (Small Bone Innovations Inc., Les Bruyères, France), led to elevated serum chrome and cobalt levels [3,10,20] and metallosis [21], causing high cup loosening rates. However, the long-term effects of polyethylene wear, which might be aggravated by the double-sided articulation of the TOUCH^®^ implant, and its impact on prosthesis stability are still not well understood [18,22,23].

To the best of our knowledge, this examination is the first user report besides the data published by the developer, Bruno Lussiez et al. [9,10,15]. Comparing our findings to the short-term ones [10] of the developer, postoperative key-pinch strengths is equivalent. Our median, postoperative pain levels as well as DASH scores slightly undercut the ones of Lussiez et al. [10]. Thus, the presents study independently corroborates the solid performance of this novel prosthesis type. Besides the TOUCH^®^ prosthesis, there is currently another dual-mobility prosthesis available, i.e., Moovis (Stryker European Holdings I, LLC) [24]. Developed in 2012, this prosthesis’s components are composed of the same material as the TOUCH^®^ prosthesis, while its design is slightly different [25]. Regarding its mid-term performance, the functional outcomes result in comparable values: DASH scores range between 12 and 35, VAS between 0 and 1, and key-pinch strength between 7 and 7.5 [24,25,26]. Moreover, failure rates between 0% and 4% might also be another indicator for the good performance of the dual-mobility mechanism.

Regarding complications, 6 out of 10 complications were tendon-related issues and 2 were due to inappropriate choice of neck size. In previous studies, several authors report prosthetic thumb CMC replacement to be challenging surgery with a steep learning curve [23]. Dumartinet-Gibaud et al. indicate that most early revision surgeries were due to technical errors, e.g., issues with trapezial reaming, cup positioning, or incorrect choice of neck size. Likewise, our two revision surgeries were also due to the latter cause. In this context, Maes-Clavier et al. even stated that an experience of 30 cases is required to accurately perform thumb CMC TJA [27]. Moreover, we hypothesize that the high incidence of tendon-related issues (de Quervain’s tendosynovitis, trigger thumb and EPL tendon rupture) are caused by the conversion of the joint’s biomechanics form a saddle to a ball and socket joint. This finding was also found by Luissez et al., who reported four cases of de Quervain’s disease and six trigger thumbs in their case series of 107 patients, and seems to be a general problem associated with thumb CMC TJA [28]. While there is a tendency in thumb CMC TJA to blame the implant design for any complications [23], the cases of EPL tendon ruptures might also be attributed to the category of technical errors. Thus, reoperations revealed that the rupture occurred at the level of the base of the thumb. Therefore, the rupture might be caused by a sharp edge resulting from resection the joint surface and shaft preparation, which gradually cut through the EPL tendon by repetitive movements of the thumb.

There are several limitations in the present study. First, our follow-up period of one year only provides short-term findings of this novel thumb CMC implant. This issue is especially important in terms of prosthesis survival. Although we could present 0 cases of cup loosening, radiolucency, or any other problems related to polyethylene wear or osteointegration, these key figures of each prosthesis type have to be observed in a longer time period to evaluate its sustainability. However, a 95% prosthesis survival rate should be deemed a beneficial result, because many publications regarding various thumb CMC prosthesis types report high failure rates within the first postoperative year [21,23,29,30]. Moreover, the failure rate of 5% might be caused by the surgeon’s learning curve more likely than by design-related issues of the prosthesis. Nevertheless, we continue to systematically follow up our patients to gather medium- and long-term data. Furthermore, the ROM outcomes have to be regarded as absolute, descriptive data. The significant, postoperative improvement in ROM values is more a statistical than a clinically relevant effect, because the median difference undercuts the ICC values resulting from reliability studies [13]. Moreover, the present study does not include a comparison cohort, consisting of e.g., trapeziectomy or resection-suspension-arthroplasty patients. Therefore, randomized-control trials have to confirm the good performance of this novel prosthesis, if long-term data confirm reliable fixation and revision rates.

## 5. Conclusions

The present study assessing the TOUCH^®^ prosthesis shows a significant improvement in DASH scores, and pain levels as well as a high survival rate, while key-pinch strength approaches that of the contralateral hand. Thus, we recommend this prosthesis type for thumb CMC stage III OA although some complications were detected related to a steep learning curve of the surgeon and tendon-associated issues due to the conversion of the joint type.

## Figures and Tables

**Table 1:** Patient demographics.

Parameter	Value
Patients	37
Thumbs	40
Age	57.7 (IQR: 13.6)
Sex (f/m)	32/8
Side (l/r)	19/21
dominant hand (l/r)	1/39

**Table 2:** Range of motion (ROM) results.

	Pre-OP	6 Months	12 Months
Thumbs (n)	40	40	40
Palmar abduction (°)	50 (10)	60 (5)	60 (10)
Radial abduction (°)	55 (10)	60 (5)	60 (5)
Opposition (cm)	0.5 (1.8)	0 (0)	0 (0)

Data are presented as median (IQR). Pre-OP: preoperative.

## Data Availability

The data that support the findings of this study are available from the first author (S.M.F.), upon reasonable request.
